# Incorporating ultrasound-based lymph node staging significantly improves the performance of a clinical nomogram for predicting preoperative axillary lymph node metastasis in breast cancer

**DOI:** 10.17305/bb.2022.8564

**Published:** 2023-08-01

**Authors:** Xiaomin Wang, Xiaoping Yi, Qian Zhang, Xiaoxiao Wang, Hanghao Zhang, Shuai Peng, Kuansong Wang, Liqiu Liao

**Affiliations:** 1 Department of Breast Surgery, Xiangya Hospital, Central South University, Changsha, Hunan, China; 2 Clinical Research Center for Breast Cancer, Xiangya Hospital, Central South University, Changsha, Hunan, China; 3 National Clinical Research Center for Geriatric Disorders, Xiangya Hospital, Central South University, Changsha, Hunan, China; 4 National Engineering Research Center of Personalized Diagnostic and Therapeutic Technology, Xiangya Hospital, Changsha, Hunan, China; 5 Hunan Engineering Research Center of Skin Health and Disease, Xiangya Hospital, Changsha, Hunan, China; 6 Department of Dermatology, Xiangya Hospital, Central South University, Changsha, Hunan, China; 7 Hunan Key Laboratory of Skin Cancer and Psoriasis, Xiangya Hospital, Changsha, Hunan, China; 8 Department of Radiology, Xiangya Hospital, Central South University, Changsha, Hunan, China; 9 Department of Pathology, Xiangya Hospital, Central South University, Changsha, Hunan, China; 10 Department of Pathology, School of Basic Medical Science, Central South University, Changsha, Hunan, China

**Keywords:** Predictive model, axillary lymph node metastasis (ALNM), breast cancer

## Abstract

Models for predicting axillary lymph node metastasis (ALNM) in breast cancer patients are lacking. We aimed to develop an efficient model to accurately predict ALNM. Three hundred fifty-five breast cancer patients were recruited and randomly divided into the training and validation sets. Univariate and multivariate logistic regressions were applied to identify predictors of ALNM. We developed nomograms based on these variables to predict ALNM. The performance of the nomograms was tested using the receiver operating characteristic curve and calibration curve, and a decision curve analysis was performed to assess the clinical utility of the prediction models. The nomograms that included clinical N stage (cN), pathological grade (pathGrade), and hemoglobin accurately predicted ALNM in the training and validation sets (area under the curve [AUC] 0.80 and 0.80, respectively). We then explored the importance of the cN and pathGrade signatures used in the integrated model and developed new nomograms by removing the two variables. The results suggested that the combine-pathGrade nomogram also accurately predicted ALNM in the training and validation sets (AUC 0.78 and 0.78, respectively), but the combine-cN nomogram did not (AUC 0.64 and 0.60, in the training and validation sets, respectively). We described a cN-based ALNM prediction model in breast cancer patients, presenting a novel efficient clinical decision nomogram for predicting ALNM.

## Introduction

Breast cancer is one of the most commonly diagnosed cancers worldwide, with over 2 million new cases in 2020 based on the GLOBOCAN [1]. Axillary lymph node (ALN) status is a meaningful indicator for clinical staging in patients with breast cancer, and it is also one of the most crucial prognostic factors, thus influencing clinical decision making [2, 3]. Axillary lymph node dissection (ALND) is the gold standard to evaluate axillary lymph node metastasis (ALNM). However, ALND is an invasive procedure that might cause operative complications [4, 5]. Sentinel lymph node biopsy (SLNB) is the current standard method for ALN staging, which determines whether or not the doctor should perform ALND and guides the surgeon’s decision for subsequent treatment [6, 7]. Unfortunately, both ALND and SLNB are invasive methods and may lead to some unacceptable complications, which would greatly reduce the quality of life of patients [8, 9]. Moreover, a long wait for SLNB results during surgery can unavoidably prolong the operation time and reduce efficiency. Therefore, there is an urgent need for a noninvasive and efficient diagnostic tool for preoperatively estimating ALNM.

Traditional noninvasive methods to confirm the ALN status are mainly preoperative imaging examinations, such as ultrasound, computed tomography (CT), and magnetic resonance imaging (MRI). However, these approaches may lead to some ALNM patients being missed due to low sensitivity [10, 11]. Previous studies have identified some risk factors for ALNM in breast cancer, but individual assessment for patients is lacking [12, 13]. With the advent of artificial intelligence, precision medicine for breast cancer has entered a bright era and new noninvasive methods have emerged to evaluate ALNM [14]. The machine learning-based approach used for evaluating the diagnosis of ALNM in breast cancer patients shows certain advantages in personalized prognostics [15]. Although several machine learning-based approaches have been used for predicting ALN status in patients, they provided little value for clinical application [16]. In addition, some imaging procedures, such as MRI and CT, are too expensive so that some patients cannot afford them. Therefore, these methods are not suitable for all patients. Hence, there is an urgent need for an accurate, efficient, clinically applicable, and extensive diagnostic method to estimate preoperative ALNM.

In the current study, we aimed to build and validate a noninvasive predictive model to accurately predict ALNM in breast cancer patients. We collected patients’ demographics and laboratory tests and extracted clinical N stage (cN) signature, which was assessed by the Breast Surgery multidisciplinary team. Then, we used clinical factors and cN to generate nomograms for preoperative prediction of ALN status in breast cancer patients to optimize decision making for personalized cancer treatment.

## Materials and methods

### Study design

**Figure 1.**
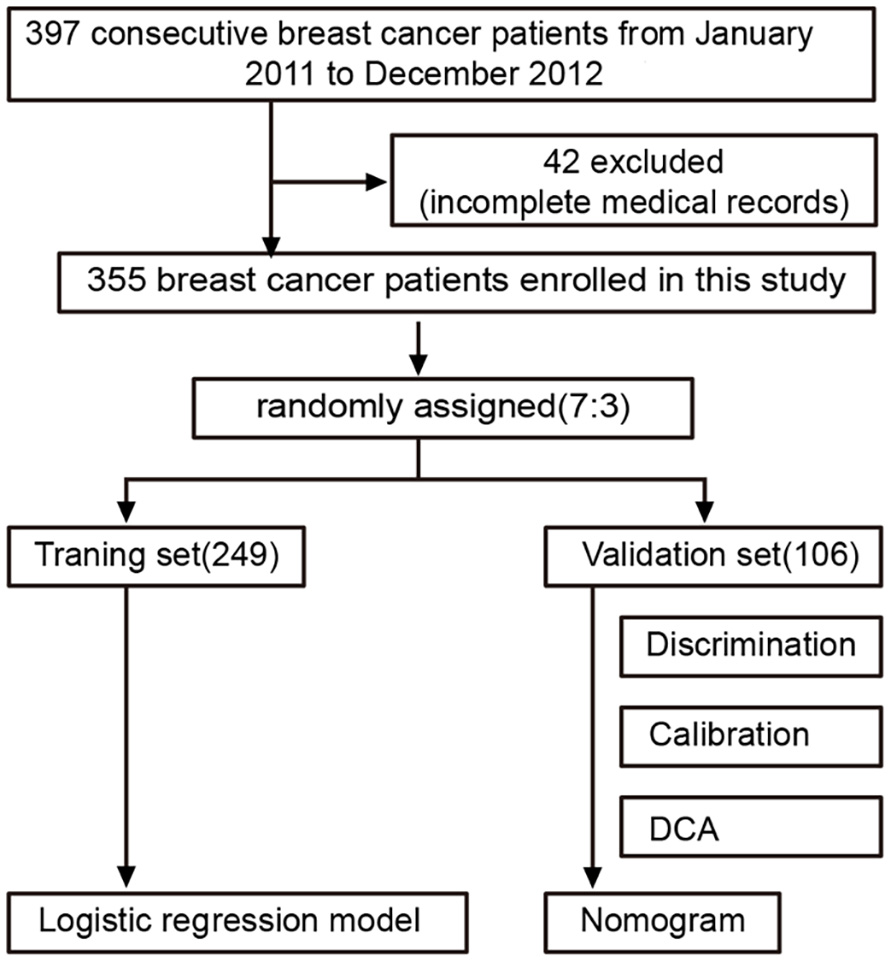
**The process of patient enrollment and nomogram development.** DCA: Decision curve analysis.

Based on the inclusion and exclusion criteria, the entire cohort ended up including 355 patients. We established and validated nomograms based on patients’ preoperative clinical characteristics and pathological features to predict ALNM in breast cancer patients. Figure 1 shows the workflow of our study.

### Study population

A total of 397 consecutive breast cancer patients treated at the Department of Breast Surgery at Xiangya Hospital (Changsha city, Hunan Province, China) from January 2011 to December 2012 were retrospectively screened, of whom 355 met the inclusion criteria and were included. The inclusion criteria were: 1) Female adults (age >18 years); 2) Histologically first confirmed breast cancer; 3) Patients who underwent ALND or SLNB to determine ALN status; 4) Patients with the absence of any distant metastasis at initial diagnosis; 5) Patients with no history of breast surgery or irradiation; 6) Patients with no other concomitant malignancy. Forty-two patients were excluded due to incomplete medical records, and a total of 355 patients were recruited in the end. The outcome was the ALN status at the surgery.

The included patients were randomly divided into two sets in a ratio of 7:3, with 249 patients in the training set and 106 patients in the validation set. The training set was used to filter the significant variables and develop nomogram, and the validation set was used to test the results obtained from the training set.

### Clinicopathologic data collection

In this study, all patients enrolled underwent palpation and breast ultrasound at diagnosis and were diagnosed with breast cancer by pathologic biopsy. Information regarding ALN status by SLNB or ALND was extracted from the records.

Data collection and analysis were conducted from December 2021 to June 2022. The privacy information of patients was protected during the research process. We did not collect information related to personal privacy, and patient identity was used only for sample coding. During the data analysis, we did not have access to patient privacy. We extracted the demographics data and laboratory parameters at diagnosis. Among these, demographics included sex, age, weight, height, and body mass index (BMI). BMI was calculated using a formula that divides weight by height into squares citation. Laboratory parameters at diagnosis included routine blood examination, electrolytes, liver function, and coagulation indices. We also collected data on TNM classification, hormone receptor status and human epidermal growth factor receptor 2 receptor (HER2) status, Ki-67, clinical and pathological staging, and tumor pathological type. Ultrasound evaluation of ALN status was performed by experienced breast radiologists, and the assessment of normal/abnormal was at the discretion of the evaluating radiologist based on ultrasound diagnostic criteria [17, 18].

TNM stage of patients was accessed by Breast Surgery multidisciplinary team according to American Joint Committee on Cancer criteria, then the clinical T stage and N stages were extracted from the patient’s preoperative TNM stage. All patients accepted SLNB or ALND. The status of the patient’s ALN was assessed according to previous reported criteria [19]. We used these variables to identify the potential independent risk factors for ALNM.

### Nomogram development

To develop a nomogram, we used a three-step approach. Univariate logistic regression was applied to identify the patient’s signature in the training set that was associated with ALNM. Second, we included the significant variables identified by univariate logistic regression with *P* < 0.05 in a multivariate logistic regression analysis to determine which factors were independent predictors of ALNM [20]. Next, the independent factors of ALNM were used to establish the nomograms for predicting ALNM, and we investigated the predictive ability of each model with and without cN or pathological grade (pathGrade).

### Ethical statement

This study was performed in line with the principles of the Declaration of Helsinki. Approval was granted by the Ethics Committee of Xiangya Hospital of Central South University (No. 202112189). Since this study was a retrospective study, the Ethics Committee waived the need to obtain informed consent from the patients.

### Statistical analysis

We used R software to randomly divide the cohort into a training set and a validation set in the ratio of 7:3, and the chi-squared test was performed to check the differences in the categorical variables between the two groups. We used the receiver operating characteristic (ROC) curve to evaluate discriminative ability and calibration plots to evaluate calibrating ability of the nomograms. Additionally, decision curve analysis (DCA) was used to quantify the clinical benefit of the cN-based nomograms. *P* < 0.05 was considered statistically significant.

## Results

### Patient characteristics

A flowchart of the study is shown in Figure 1. A total of 355 breast cancer patients meeting the requirements from January 2011 to December 2012 were enrolled in this study. Tables 1 and 2 show the clinical characteristics of the whole set (355 patients), the training set (249 patients) and the validation set (106 patients). According to the result of ALND, ALNM was confirmed in 157 (44.23%) patients, 110 (44.18%) in the training set and in 47 (44.34) in the validation set. According to American Joint Committee on Cancer TNM staging system, clinical stages I, II, and III accounted for 11.3%, 67.0%, and 21.7%, respectively, in the entire set; 20.48%, 60.64%, and 18.87%, respectively, in the training set, and 13.20%, 66.98%, and 17.92%, respectively, in the validation set.

**Table 1:** Clinicopathologic characteristics of patients in the entire, training, and validation sets

	***n* (%)**			
	**Entire set**	**Training set**	**Validation set**	
**Characteristic**	**(*n* ═ 355)**	**(*n* ═ 249)**	**(*n* ═ 106)**	***P* value**
*Age (years)*				0.171
<50	223 (62.82)	150 (60.24)	72 (67.92)	
≥50	132 (37.18)	99 (39.76)	34 (32.08)	
*Clinical T stage*				0.386
1	73 (20.56)	57 (22.89)	16 (15.09)	
2	218 (61.41)	147 (59.04)	71 (66.98)	
3	42 (11.83)	30 (12.05)	12 (11.32)	
4	22 (6.20)	15 (6.02)	7 (6.6)	
*Clinical N stage*				0.035*
0	216 (60.85)	162 (65.06)	54 (50.94)	
1	99 (27.89)	60 (24.1)	39 (36.79)	
2	37 (10.42)	24 (9.64)	13 (12.26)	
3	3 (0.85)	3 (1.2)	0 (0)	
*Clinical TNM stage*				0.205
I	65 (18.31)	51 (20.48)	14 (13.20)	
II	222 (62.5)	151 (60.64)	71 (66.98)	
III	68 (19.15)	47 (18.87)	21 (17.92)	
*Pathological TNM stage*				0.45
I	40 (11.27)	31 (12.45)	9 (8.49)	
II	238 (67.04)	167 (67.07)	71 (66.98)	
III	77 (21.69)	51 (20.48)	26 (24.53)	
*ER status*				0.654
Negative	103 (29.01)	74 (29.72)	29 (27.36)	
Positive	77 (70.99)	175 (70.28)	77 (72.64)	
*PR status*				0.981
Negative	141 (39.72)	99 (39.76)	42 (39.62)	
Positive	214 (60.28)	150 (60.24)	64 (60.38)	
*HER2*				0.788
Negative	251 (70.70)	175 (70.28)	76 (71.70)	
Positive	104 (29.30)	74 (29.72)	30 (28.30)	
*p53*				0.62
Negative	104 (29.30)	71 (28.51)	33 (31.13)	
Positive	251 (70.70)	178 (71.49)	73 (68.87)	
*Ki-67*				0.766
≤20%	254 (71.55)	177 (71.08)	77 (72.64)	
>20%	101 (28.45)	72 (28.92)	29 (27.36)	

*Fisher’s precision probability test TNM: Tumor-node-metastasis; ER: Estrogen receptor; PR: Progesterone receptor; HER2: Human epidermal growth factor receptor 2; Ki67: Proliferation marker protein Ki67.

**Table 2:** Clinical characteristics of patients with or without axillary lymph node metastasis across entire, training, and validation sets

	***n* (%), by set and axillary lymph node status**								
	**Entire set (*n* ═ 355)**			**Training set (*n* ═ 249)**			**Validation set (*n* ═ 106)**		
**Characteristic**	**Negative (*n* ═ 198)**	**Positive (*n* ═ 157)**	***P* value**	**Negative (*n* ═ 139)**	**Positive (*n* ═ 110)**	***P* value**	**Negative (*n* ═ 59)**	**Positive (*n* ═ 47)**	***P* value**
*Age (years)*			0.534			0.217			0.42
<50	121 (61.11)	101 (64.33)		79 (56.84)	71 (64.55)		42 (71.19)	30 (63.83)	
≥50	77 (38.89)	56 (35.67)		60 (43.17)	39 (35.45)		17 (28.81)	17 (36.17)	
*Clinical T stage*			<0.001			<0.001			0.003*
1	55 (27.78)	18 (11.46)		40 (28.78)	17 (15.45)		15 (25.42)	1 (2.13)	
2	123 (62.12)	95 (60.51)		86 (61.87)	61 (55.45)		37 (62.71)	34 (72.34)	
3	14 (7.07)	28 (17.83)		9 (6.47)	21 (19.09)		5 (8.47)	7 (14.89)	
4	6 (3.03)	16 (10.19)		4 (2.88)	11 (10)		2 (3.39)	5 (10.64)	
*Clinical N stage*			<0.001*			<0.001*			<0.001
0	163 (82.32)	53 (33.76)		120 (86.33)	42 (38.18)		43 (72.88)	11 (23.4)	
1	31 (15.66)	68 (43.31)		16 (11.51)	44 (40)		15 (25.42)	24 (51.06)	
2	4 (2.02)	33 (21.02)		3 (2.16)	21 (19.09)		1 (1.69)	12 (25.53)	
3	0 (0)	3 (1.91)		0 (0)	3 (2.73)		0 (0)	(0)	
*Clinical TNM stage*			<0.001			<0.001*			<0.001*
I	52 (26.26)	13 (8.28)		38 (27.34)	13 (11.82)		14 (23.73)	0 (0)	
II	134 (67.68)	88 (56.05)		94 (67.63)	57 (51.82)		40 (67.8)	31 (65.96)	
III	12 (6.06)	56 (35.67)		7 (5.04)	40 (36.36)		5 (8.47)	16 (34.04)	
*Pathological TNM stage*			<0.001			0.002			0.175*
I	33 (16.67)	7 (4.46)		26 (18.71)	5 (4.55)		7 (11.86)	2 (4.26)	
II	131 (66.16)	107 (68.15)		90 (64.75)	77 (70)		41 (69.49)	30 (63.83)	
III	34 (17.17)	43 (27.39)		23 (16.55)	28 (25.45)		11 (18.64)	15 (31.91)	
*ER status*			0.284			0.452			0.512
Negative	62 (31.31)	41 (26.11)		44 (31.65)	30 (27.27)		18 (30.51)	11 (23.4)	
Positive	136 (68.69)	116 (73.89)		95 (68.35)	80 (72.73)		41 (69.49)	36 (76.6)	
*PR status*			0.72			0.555			0.803
Negative	77 (38.89)	64 (40.76)		53 (38.13)	46 (41.82)		24 (40.68)	18 (38.3)	
Positive	121 (61.11)	93 (59.24)		86 (61.87)	64 (58.18)		35 (59.32)	29 (61.7)	
*HER2*			0.347			0.519			0.461
Negative	144 (72.73)	107 (68.15)		100 (71.94)	75 (68.18)		44 (74.58)	32 (68.09)	
Positive	54 (27.27)	50 (31.85)		39 (28.06)	35 (31.82)		15 (25.42)	15 (31.91)	
*p53*			0.482			0.217			0.564
Negative	61 (30.81)	43 (27.39)		44 (31.65)	27 (24.55)		17 (28.81)	16 (34.04)	
Positive	137 (69.19)	114 (72.61)		95 (68.35)	83 (75.45)		42 (71.19)	31 (65.96)	
*Ki-67*			0.269			0.429			0.415
≤20%	137 (69.19)	117 (74.52)		96 (69.06)	81 (73.64)		41 (69.49)	36 (76.6)	
>20%	61 (30.81)	40 (25.48)		43 (30.94)	29 (26.36)		18 (30.51)	11 (23.4)	

*Fisher’s precision probability test TNM: Tumor-node-metastasis; ER: Estrogen receptor; PR: Progesterone receptor; HER2: Human epidermal growth factor receptor 2; Ki67: Proliferation marker protein Ki67.

### Signature screening and nomogram construction

**Figure 2.**
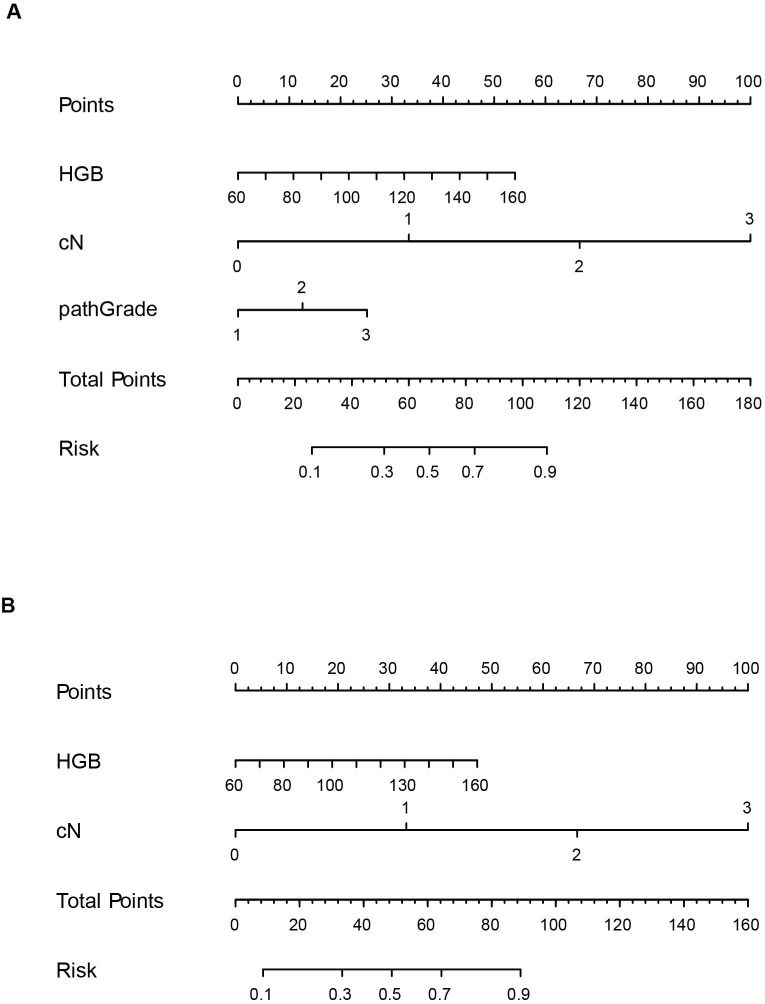
**Nomograms for predicting axillary lymph node metastasis based on risk factors.** (A) Nomogram incorporating the HGB, cN, and pathGrade. (B) Nomogram including HGB and cN. HGB: Hemoglobin; cN: Clinical N stage; pathGrade: Pathological grade.

The training set contained 249 patients, of whom 110 (44.18%) patients had ALNM. Univariate and multivariate logistic regression analyses were used to identify the independent risk factors for ALNM in breast cancer patients (Tables 3 and S1). We found that hemoglobin (odds ratio [OR] 1.03, 95% confidence interval [CI] 1.00–1.06), cN (OR 5.32, 95% CI 3.05–9.26), and pathGrade (OR 1.93, 95% CI 1.13–3.30) were independent predictors of ALNM (Table 3). Then, we constructed a nomogram using all these independent predictors for ALNM whose *P* < 0.05 (Figure 2A). Finally, we explored the importance of the cN and pathGrade included in the integrated model and developed a new nomogram by removing the pathGrade variable (Figure 2B).

**Table 3:** Multivariate logistic regression analyses of axillary lymph node metastasis

**Variables**	**OR**	**95% CI**	***P* value**
HGB	1.03	1.00–1.06	0.04698
cT	1.39	0.91–2.11	0.12357
cN	5.32	3.05–9.26	<0.0001
pathGrade	1.93	1.13–3.30	0.01646

CI: Confidence interval; OR: Odds ratio; HGB: Hemoglobin; cT: Clinical T stage; cN: Clinical N stage; pathGrade: Pathological grade.

### Nomogram validation

**Figure 3.**
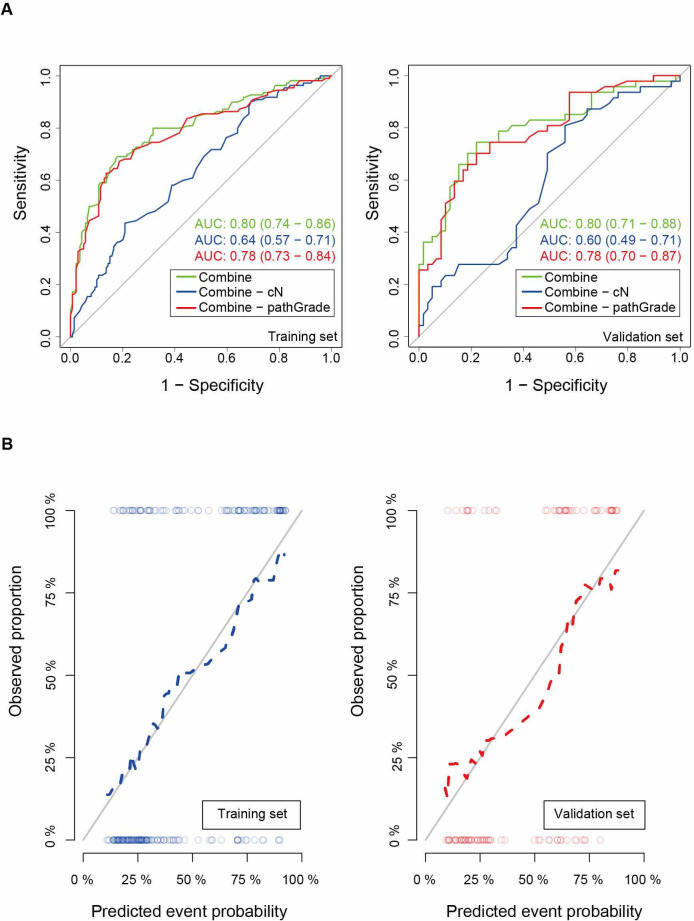
**ROC and calibration curves of the preoperative prediction of axillary lymph node metastasis in breast cancer patients.** (A) The ROC curves of different nomograms with AUC for the training set (left) and the validation set (right). (B) Calibration curves of the combine model for the training set and validation set. ROC: Receiver operating characteristic; AUC: Area under the curve.

**Figure 4.**
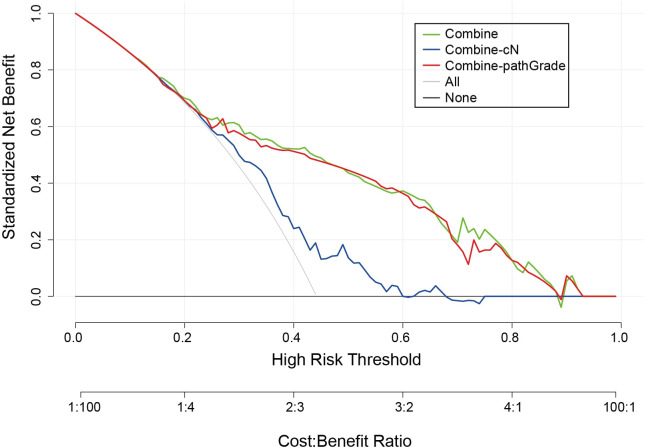
**Decision curve analysis for the ALNM-nomograms in the training set.** Net benefit is shown on the *Y*-axis. cN: Clinical N stage; pathGrade: Pathological grade; ALNM: Axillary lymph node metastasis.

We performed ROC analysis on the two sets using different models. Both models highlighted satisfactory accuracy in predicting the probability of ALNM. The area under the curve (AUC) of the ROC curves (Figure 3A) showed valuable discriminative ability for predicting ALNM in the combined model in the training set (AUC 0.80, 95% CI 0.74–0.86) and in the validation set (AUC 0.80, 95% CI 0.71–0.88). When we removed the pathGrade variable and developed a new model named combine-pathGrade to investigate whether pathGrade was an important risk factor in the combine model, similarly, the new model showed good predictive ability in the training set (AUC 0.78, 95% CI 0.73–0.84) and in the validation set (AUC 0.78, 95% CI 0.70–0.87). In addition, to clarify the importance of cN for predicting ALNM, we developed a new model named combine-cN by removing the cN variable from the combine model. The ROC curve of the combine-cN model showed that the discriminative ability decreased significantly, with the AUCs of 0.64 (95% CI 0.57–0.71) in the training set and 0.60 (95% CI 0.49–0.71) in the validation set. Importantly, our results suggested that the cN played a dominant role in predicting ALNM. The calibration curve (Figure 3B) showed that the predicted and observed probability of ALNM were in good agreement. Meanwhile, the DCA exhibited great clinical benefits for predicting ALNM. When the threshold probability was > 15% in the training set, using the combine model and combine-pathGrade model added more benefits than the treat-all-patients scheme or the treat-none scheme in predicting ALNM in breast cancer patients (Figure 4). Actually, the combine and combine-pathGrade models showed better results than the combine-cN model in predicting ALNM.

Therefore, our nomograms show a good ability in forecasting the probability of ALNM.

## Discussion

The global incidence of breast cancer is increasing rapidly, and it is of great clinical significance to study the diagnostic prediction of breast cancer [21]. ALN status of breast cancer patients affects their prognosis and also affects doctors’ decision on treatment options. The misjudgment of the ALNM may lead to inappropriate treatment of the patients [22]. At the time of the initial diagnosis of breast cancer, ALNM predicts poor future treatment outcomes in breast cancer patients. Studies have reported a worse prognosis in ALN-positive patients than in ALN-negative patients, so an accurate assessment of the status of ALN in breast cancer patients before treatment can optimize treatment strategies and improve outcomes [23, 24]. Therefore, it is of great interest to clarify the ALN status of breast cancer patients at initial diagnosis.

Most previous research on ALNM in breast cancer patients has only focused on single independent risk factors for ALNM, such as tumor size and grade [25, 26]. Recently, a number of researchers have also developed multivariate models to predict ALNM based on patient clinical information [25, 27]. The greatest advantage we have over these studies is that our model is noninvasive and convenient. There have also been attempts to investigate the relationship between the tumor immune microenvironment and ALNM. A previous study used the tumor-infiltrating lymphocytes signature to predict ALNM status in breast cancer patients, but in their study, few HER2-positive breast cancer and triple negative breast cancer patients were included. In addition, they only focused on T1 breast cancer [28]. Some researchers investigated the use of MRI radiomic signature to develop a noninvasive preoperative model for predicting ALNM [15, 29]. Liu et al. [30] used the MRI radiomic signature to predict ALN status, however, the sample size of this single-center study was too small. In the present study, we enrolled a larger cohort including 355 breast cancer patients to develop nomograms. Our approach can be extended to a variety of clinical and experimental applications. Yao et al. used MRI radiomics signature to develop and validate a model for predicting ALNM and disease-free survival in patients with early-stage breast cancer. Their study showed that the predictive model combining radiological and clinical information for predicting ALNM was better than a model using either one alone [15]. However, in this multicenter retrospective study, there was heterogeneity in the magnetic resonance versions. Similarly, another retrospective study demonstrated that preoperative internal enhancement on dynamic contrast-enhanced MRI might help predict sentinel lymph node metastasis in patients with invasive breast cancer [29]. But the indicator was subjective because it was set by the radiologists, and as little is known about the reproducibility of measurements, the reproducibility of this method is uncertain. Unfortunately, the cost of MRI is relatively high, and the availability of MRI units in primary hospitals remains poor. Compared with those studies using MRI radiomic signature to predict ALNM status, our nomograms are much easier and simpler to perform, and the data needed in this model is easier and more convenient to be obtained.

Unlike previous studies that predicted ALNM, our study cohort included clinical lymph node staging of patients and other information to predict ALN status, resulting in a more accurate preoperative assessment of ALN status [30, 31]. In this study, we developed and validated models that can simply and accurately predict ALNM status based on patient clinical information at a baseline level. Furthermore, we found that among these models, cN signature is a crucial factor for predicting ALNM in patients with breast cancer. Our models displayed an excellent ability to predict ALNM with good AUCs in the combine validation set (AUC 0.80, 95% CI 0.71–0.88) and the combine-pathGrade validation set (AUC 0.78, 95% CI 0.73–0.84). In addition, we constructed different nomograms to distinguish which signature plays the dominant role in the predictive model. When we removed the cN variable, the discriminative ability for predicting ALNM of the ROC curve decreased significantly, with the AUCs of 0.64 (95% CI 0.57–0.71) in the training set and 0.60 (95% CI 0.49–0.71) in the validation set. However, when we removed the pathGrade variable, the combine-pathGrade model still showed great discriminative ability for predicting ALNM. Our results suggested that cN played a dominant role in predicting ALNM, and nomograms, including cN, showed good abilities in predicting ALNM in breast cancer patients. In line with our study, previous studies showed that ALN condition based on breast ultrasound detection was a predictor of lymph node load [32]. However, in another research, the accuracy of the model using axillary ultrasound was not high, with an AUC of 0.585–0.719 [33]. Compared to these previous studies, our nomograms showed good predictive abilities. A previous study demonstrated that tumor lesion boundary, tumor size, and tumor quadrant locations were the most important factors affecting ALNM in cT1-2N0M0 stage breast cancer, and we would like to focus on these risk factors in our further study [34]. Besides, previous studies have focused on the predictive role of imaging features on ALNM, but little focus has been given to laboratory test indicators. In the present study, our results suggest that hemoglobin is a risk factor for ALNM, which has not been previously reported in studies, suggesting that an understanding of the laboratory parameters of patients helps predict ALN status. In the follow-up study, we plan to explore whether biochemical indicators, such as blood lipids and blood glucose, can predict the status of lymph node metastases in breast cancer patients.

Admittedly, this study has several limitations. First, our models were built based on data collected from a single center, and because this is a retrospective study, its clinical applicability may be reduced. Multicenter evidence will be needed to validate the models in the future before they can be put into clinical use. Second, we did not conduct subgroup analyses due to the small sample size. The possibility of ALNM may differ among breast cancer patients with different molecular subtyping. In future study, we will assess the ability of the predictive models to predict ALNM in each molecular subtype. Additionally, the cN relies heavily on the ultrasound results and the expertise of the doctors, which is subjective and little is known about the reproducibility of those methods. Fourth, due to medical limitations at the time of the patient’s diagnosis approximately 10 years ago and the lack of genetic testing data, we focused only on the potential impact of clinical and pathological features on ALNM and did not consider the genetic features. For future prospective studies, researchers may combine transcriptome and gene mutation data for predicting ALNM. Finally, we have validated the effects of our nomograms by using the validation set which may overestimate the value of our model, and prospective external validation is lacking.

## Conclusion

This study described a cN-based prediction model for ALNM in breast cancer patients, presenting a novel personalized clinical decision nomogram that can be used to predict ALNM status. The integrated nomogram is valuable for determining preoperative ALNM. When removing the pathGrade signature based on puncture pathology results from the integrated model, the nomogram did not reduce the predictive accuracy of the nomogram. But removing cN signature significantly reduced the predictive ability of the prediction model. Our results suggest that cN staging based on preoperative ultrasound is valuable for determining preoperative ALNM. The cN-based nomograms are useful clinical tools for predicting ALNM and can provide a preoperative prediction.

## Supplemental Data

**Table S1:** Univariate logistic regression analyses of axillary lymph node metastasis

**Variables**	**OR**	**95% CI**	***P* value**
Age	0.98	0.95–1.00	0.08
Weight	1.00	0.96–1.03	0.85
Height	0.98	0.94–1.03	0.49
BMI	1.01	0.93–1.09	0.90
Neu	1.10	0.90–1.34	0.34
Lym	0.86	0.53–1.42	0.56
NLR	1.10	0.88–1.38	0.39
PLT	1.00	0.99–1.00	0.51
PLR	1.00	0.99–1.01	0.82
Mono	3.40	0.65–17.69	0.15
LMR	0.95	0.83–1.10	0.50
A	0.96	0.90–1.03	0.27
G	1.01	0.96–1.07	0.61
AG	0.51	0.19–1.35	0.17
AST	1.01	0.97–1.05	0.63
ALT	1.01	0.99–1.03	0.45
LDH	1.00	0.99–1.00	0.23
HGB	1.03	1.00–1.05	0.03
HCT	1.01	0.97–1.04	0.71
Eos	0.36	0.03–4.11	0.41
Bas	0.06	0.00–13.97	0.31
PCT	2.05	0.02–176.35	0.75
MPV	1.08	0.94–1.24	0.27
PDW	1.00	0.82–1.22	0.99
BUN	0.99	0.82–1.20	0.91
Scr	0.99	0.97–1.01	0.53
K	1.32	0.65–2.67	0.44
Na	0.94	0.85–1.05	0.28
Cl	0.98	0.93–1.03	0.36
CO2	0.96	0.87–1.06	0.39
AG	1.01	0.93–1.09	0.87
Ca	0.23	0.02–2.15	0.20
P	0.66	0.18–2.41	0.53
Mg	0.33	0.00–21.44	0.60
PT	1.25	0.85–1.85	0.25
PPT	0.99	0.97–1.01	0.19
INR	12.29	0.26–584.25	0.20
APTT	1.08	1.00–1.17	0.06
TT	0.96	0.79–1.16	0.67
FIB	0.91	0.62–1.32	0.61
cT	2.07	1.44–2.98	<0.001
cN	6.10	3.60–10.36	<0.001
pathGrade	2.11	1.32–3.37	<0.001
ER	0.99	0.77–1.26	0.92
PR	0.87	0.66–1.14	0.31
Ki67	0.80	0.46–1.39	0.43
P53	1.24	0.87–1.77	0.24
HER2	1.22	0.79–1.86	0.37

CI: Confidence interval; OR: Odds ratio; Neu: Neutrophil; Lym: Lymphocyte; NLR: Neutrophil to lymphocyte ratio; PLT: Platelets; PLR: Platelet to lymphocyte ratio; Mono: Monocyte; LMR: Lymphocyte to monocyte ratio; A: Albumin; G: Globulin; A/G: Albumin to globulin ratio; AST: Aspartate transaminase; ALT: Alanine transaminase; LDH: Lactate dehydrogenase; HGB: Hemoglobin; HCT: Hematocrit; Eos: Eosinophil; Bas: Basophil; PCT: Thrombocytocrit; MPV: Mean platelet volume; PDW: The distribution width of platelets; BUN: Blood urea nitrogen; Src: Serum creatinine; CO2: Carbon dioxide; AG: Anion gap; PT: Prothrombin time; PPT: Percentage of prothrombin; INR: International normalized ratio; APTT: Activated partial thromboplastin time; TT: Thromboplastin time; FIB: Fibrinogen; cT: Clinical T stage; cN: Clinical N stage; pathGrade: Pathological grade; ER: Estrogen receptor; PR: Progesterone receptor; Ki67: Proliferation marker protein Ki67; HER2: Human epidermal growth factor receptor 2.
